# AI-Driven Data Extraction and AGREE-Based Greenness Evaluation of Analytical Methods for Natural Products: Insights from Selected Studies

**DOI:** 10.1021/acs.analchem.6c02529

**Published:** 2026-07-29

**Authors:** Paweł Świt, Fabian Hammerle, Markus Ganzera

**Affiliations:** † Institute of Chemistry, Faculty of Science and Technology, University of Silesia in Katowice, Szkolna 9, Katowice 40-006, Poland; ‡ Institute of Pharmacy, Department of Pharmacognosy, 27255 University of Innsbruck, Innrain 80-82, Innsbruck 6020, Austria

## Abstract

Large language models (LLMs) have evolved into versatile tools of the 21st century, simplifying repetitive and labor-intensive tasks in everyday life. Here, we aimed to test the feasibility of using different LLMs to assess the greenness of analytical procedures according to the “Analytical GREEnness Metric Approach” (AGREE) by extracting specific data corresponding to the 12 principles of green analytical chemistry from scientific articles. Seven open-access articles on plant natural products using different analytical techniques were evaluated with the five most popular artificial intelligence (AI) tools (ChatGPT, Copilot, Perplexity, Claude, and Gemini), which were tasked to obtain specific data, along with a justification for the selection of this data. Additionally, different versions (basic and advanced) of the same tool (Perplexity and Perplexity Pro), output types (PDF file and link to the online version), and repeatability (three times the same task) were compared. All extracted data were used to calculate AGREE scores, and the final results were compared with those obtained by experts. AI tools were able to assess greenness with a high degree of accuracy, similar to that of trained researchers. Furthermore, a verification/comparison study demonstrated the possibility of critically examining the greenness assessment of developed methods to facilitate standardizing greenness evaluation. The final application of advanced AI tools dedicated to scientific research confirmed the greenness scores, indicating high consistency between all considered evaluation methods.

## Graphical abstract

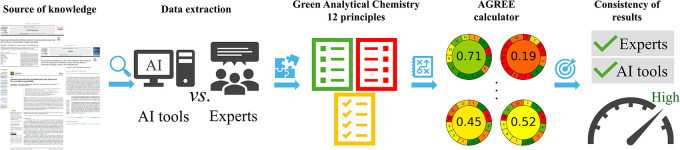

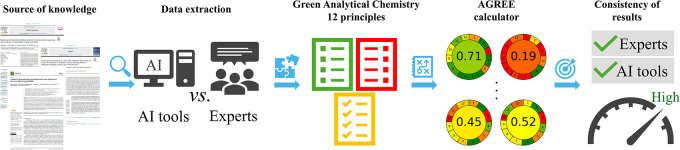

## Introduction

The concept of green analytical chemistry (GAC), derived from the ideas of green chemistry, green chemical engineering, and green chemical technology, is a key part in contemporary analytical chemistry.^1–4^ GAC aims to reduce the negative impact of chemical analyses on the environment as well as on the health and lives of humans and animals. The methods should favor miniaturization, automation, shorter analysis times, direct analysis techniques, and fewer procedure steps, from sample collection through all procedural steps to measurement. At the same time, it is essential to ensure the quality and reliability of the obtained results, as well as their environmental friendliness. This also ensures maximum compliance with sustainable development goals (i.e., meeting the needs of current generations without compromising the ability of future generations to meet their own needs). GAC is based on 12 principles proposed in 2013,^5^ which clearly and unambiguously gather and specify the aspects crucial for ensuring high greenness of analytical procedures. Additionally, the SIGNIFICANCE parameter (as a final result describing greenness) was introduced. This parameter refers to the degree of significance of a specific parameter (or indicator) in assessing “greenness” and relates to the overall assessment of a given method. The higher this parameter’s value, the greater the compliance with the GAC principles, the environmental friendliness, and the safety of the procedure. The enormous development in this area, along with the innovations, underscores the importance of the concept of GAC in the contemporary world.^6,7^ Sustainable development requires optimized chemical analytics to enable monitoring and management of environmental impact of the analytical methods.

To date, several metrics and tools have been developed to assess chemical procedures in terms of environmental friendliness, allowing for comparisons between methods and assessment of their safety.^8–12^ Recent years have seen an ever-growing interest in the development and proposal of various tools for assessing procedures across a range of comprehensive aspects.^13–18^ Numerous review articles have described individual tools used to evaluate analytical procedures^19–25^ and to guide assessments.^26,27^ However, only a few tools provide a complete and detailed characterization of analytical methods in the context of greenness in relation to the 12 GAC principles. One such tool is the Analytical GREEnness Metric Approach (AGREE),^28^ proposed in 2020. It converts the greenness score to a 0–1 scale. Additionally, each of the 12 principles can be interpreted separately, clearly identifying areas requiring improvement. A color scale (from green to red) visually highlights the evaluation process. The analysis is performed using open-source software and includes detailed reporting. This is by far the most popular tool (2,983 citations, as of March 30, 2026).

The White Analytical Chemistry (WAC) concept is also worth noting, which extends GAC and incorporates practical elements and those related to analytical method validation.^29,30^ WAC refers to the Red-Green-Blue (RGB) model^31^ and encompasses three different aspects: analytical performance (red), compliance with the “green” chemistry principles (green), and productivity/practical effectiveness (blue). This concept classifies an analytical method into one of nine base colors. If the analytical procedure meets all the criteria, it is classified as white.

Besides, the number of scientific articles on the development of new analytical methods also continues to increase each year. Data on sample preparation, instrument combinations, method parameters, and validation are presented in various forms (tables, figures, text) and across different sections of the articles, making it difficult to extract the necessary information for assessing greenness. At the same time, artificial intelligence has become an integral part of our everyday lives, where especially large language models such as ChatGPT can have the most diverse applications. As a consequence, AI chatbots could enable rapid extraction of data needed to assess greenness. The use of AI tools in evaluating analytical methods should enable automated large-scale data extraction and reduce human work. This could improve transparency and repeatability, key factors that ensure adequate resource allocation and pollution reduction. Additionally, this would facilitate the construction of appropriate databases containing the most important parameters of developed analytical methods, along with an assessment of their environmental friendliness in a subjective and unified manner. The use of AI tools has both advantages and disadvantages in data extraction and greenness assessment.^32^ However, the use of AI in analytical chemistry,^33–35^ method planning,^36^ and greenness assessment^37–40^ is becoming increasingly common. With appropriately formulated tasks, such tools could be useful for extracting high-quality data needed for evaluating analytical procedures. This may be another promising direction for AI in various aspects of chemistry and beyond.^41–44^ For example, LLMs have been used to assess chemical knowledge and reasoning skills relative to experts,^45^ to rethink chemical research,^46^ for predictive chemistry,^47,48^ and to answer research questions in chemistry.^49–51^ AI has been used in modeling pollution dynamics and sustainable remediation.^52^ All these examples indicate the vast potential of AI applications in science, along with numerous benefits,^53^ which aligns with the growing emphasis on sustainable development across research disciplines.^54^

The main goal of this study is to examine the feasibility of using publicly available general-purpose AI tools, as well as advanced AI systems specifically designed for scientific research, to extract key data for assessing the greenness of analytical procedures described in published articles, compared to expert assessment. Such analysis allows for a quicker and more objective (true, unbiased, fact-based and easy to verify) evaluation of the developed methods compared to the assessment conducted by the authors of the given work. The AGREE tool was selected for the greenness assessment because it is highly popular and has been widely used in recent years, and simultaneously incorporates all 12 GAC principles. The sustainable challenge of modern analytics requires an AI framework that is consistent with several sustainable development principles (SDG) (SDG 9: Industry, innovation and infrastructure; SDG 12: Responsible consumption and production, SDG 13: Climate action)—such an approach revolutionizes analytical chemistry toward sustainable analytics. The presented study is interdisciplinary, combining analytical chemistry, data science/AI, and sustainable development science, and provides tools for both scientific research and society. This article presents a new perspective on using freely available AI chatbots for specific tasks, a practice already common in laboratories worldwide, and highlights their potential to unlock new possibilities in analytical chemistry.^34^

## Methods

### Research Scheme

The research design consisted of analyzing selected scientific articles (Articles 1–7)^55–61^ and extracting data useful for assessing greenness (AGREE) using AI tools in combination with an optimized prompt (Table S1), thereby considering several aspects (Figure 1). The first and most crucial one was testing the five AI chatbots ChatGPT, Copilot, Perplexity, Claude, and Gemini for data extraction. This allowed for comparison of results between different models. The next goal was to test different versions of a given AI tool. Two versions of Perplexity were compared: the standard (free/basic) publicly available version and the Pro (paid/advanced) version with access to advanced LLMs. In all cases, the source of data was the article PDF, which was uploaded along with a detailed description of the task. For this reason, it was decided to compare results for the source file type (PDF or an online article) (analyses were performed using Perplexity Pro). A crucial aspect was verifying the repeatability of the results, and the Perplexity Pro version was used for this purpose, given its ability to perform a larger number of analyses and the lack of limitations encountered with free versions of other AI tools. The same task (with a PDF source file) was performed three times for each article. The selected articles were also evaluated by experienced researchers in the topics covered by these articles (Experts) to verify the data extracted by the AI tools. In the verification/comparison phase, both the free and the Pro versions were used. Furthermore, five cases (Cases 1–5)^62–65^ were selected, where the study authors included the determination of AGREE scores to evaluate their procedures. The goal of this phase was to compare the authors’ results with those obtained through AI data extraction.

**Figure 1:**
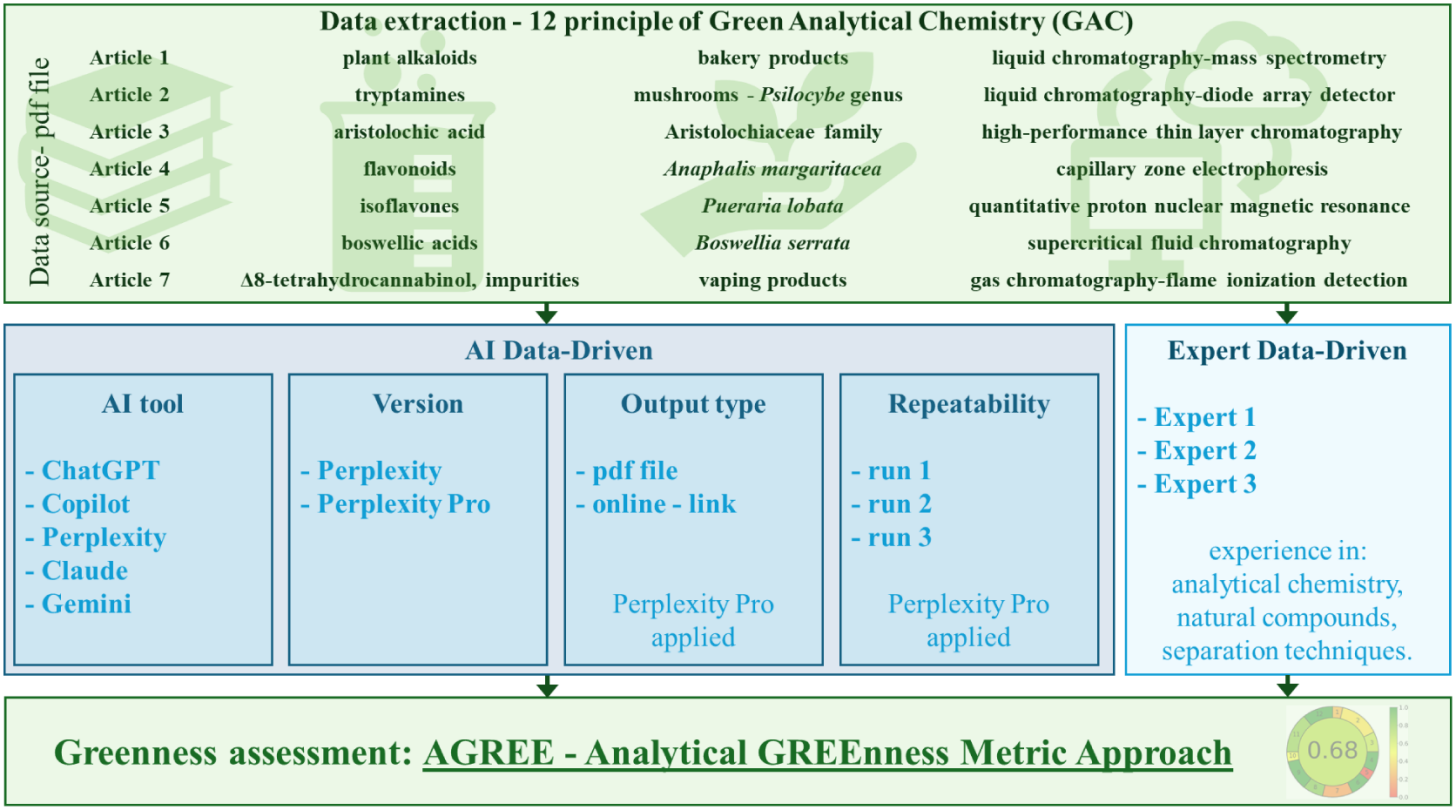
Study design illustrated as a diagram. Key data necessary for AGREE assessment were extracted from selected literature sources using different AI tools. Results were compared to those obtained by established experts.

### Articles Used for Testing and Verification/Comparison

To conduct all data extraction tests (AI vs Experts), seven articles were selected, thematically related to the use of various analytical techniques for characterizing natural products of plant origin (Figure 1). All articles were published in reputable scientific journals from the Journal Citation Reports (JCR) list with open access (this allows for analyses using AI from a legal perspective) (Table S2). Additionally, during the verification/comparison phase, four scientific articles with reported AGREE analysis were selected, and compared to the results obtained through data extraction using AI tools and the authors’ own scores (Table S2).

### AGREE Tool Analysis and Tool Description

To assess greenness based on the extracted data, the most popular tool in recent years the Analytical GREEnness Metric Approach (AGREE), proposed in 2020, was employed. First, the results from both the locally installed software (https://mostwiedzy.pl/AGREE) and the online version (agree-index.anvil.app) were compared. The results obtained in both cases were the same, but in the first case, it was possible to generate not only pictograms but also detailed reports, which were also used for analysis.

The AGREE calculator evaluates greenness against 12 principles of green analytical chemistry, and the final parameter, SIGNIFICANCE, is converted to a scale of values from 0 to 1. The following rules are considered:

– Rule 1. Direct analytical techniques should be applied to avoid sample treatment.

– Rule 2. Minimal sample size and minimal number of samples are goals.

– Rule 3. In situ measurements should be performed.

– Rule 4. Integration of analytical processes and operations saves energy and reduces the use of reagents.

– Rule 5. Automated and miniaturized methods should be selected.

– Rule 6. Derivatization should be avoided.

– Rule 7. Generation of a large volume of analytical waste should be avoided and proper management of analytical waste should be provided.

– Rule 8. Multianalyte or multiparameter methods are preferred versus methods using one analyte at a time.

– Rule 9. The use of energy should be minimized.

– Rule 10. Reagents obtained from renewable source should be preferred.

– Rule 11. Toxic reagents should be eliminated or replaced.

– Rule 12. The safety of the operator should be increased.

The final result is a pictogram representing the overall score on a 0–1 scale, located in the center, with colors (on a red-yellow-green scale) denoting the outcome. The closer the values are to 1, the greener the procedure is, which is also indicated by the dark green color. Additionally, each criterion is assigned a color along with a number and a weight (1–4). The width of the corresponding segments in the pictogram can determine the weight of each principle. Depending on the research objective and the analytical system, individual criteria may have different meanings, making it extremely important to assign weights to them accordingly.

The data extracted from selected articles (Articles 1–7) and cases for verification/comparison (Cases 1–5) using AI tools, as well as obtained after expert analysis, were used for evaluation with the AGREE tool. The number of articles selected for this proof-of-concept analysis is sufficient and representative, given the diversity of analytical techniques used to analyze natural products (the authors’ areas of expertise, which ensure expert analysis). This allows for a comprehensive evaluation of AI strategies without diluting the results with broader themes, thereby enabling a detailed analysis of the differences between AI and experts. A larger scale analysis (hundreds of articles) will be conducted after implementing the planned application programming interface (API) automation.

### AI- and Expert-Driven Data Extraction

Five of the most popular and widely available AI tools were used to extract data from published studies. These were the following tools: ChatGPT (OpenAI, San Francisco, USA), Copilot (Microsoft, Redmond, USA), Perplexity (Perplexity AI, San Francisco, USA), Claude (Anthropic, San Francisco, USA), and Gemini (Google DeepMind/Google, Mountain View, USA). Additionally, the Pro version of one of the tools, Perplexity Pro (Perplexity AI), was tested. All analyses were performed in October and November 2025. Four advanced AI tools dedicated to scientific research were used to compare the final results obtained using available methods (experts, article authors, and publicly available AI tools). This group included the following tools: Elicit (Ought, San Francisco, USA), Paperguide (Paperguide, New York, USA), SciSpace (SciSpace, Bengaluru, India), and Consensus (Consensus, Boston, USA). These analyses were conducted in May 2026 via their official web interfaces. The default model configuration offered to the user at the time was used. Manual model selection was not performed, and all results refer to the default and best model for the specific time periods in which the analyses were conducted. Due to the LLMs’ constant updates, this approach reflects real-world usage conditions (which is also the goal of this article). However, it does not allow for full reproduction of the exact model versions under which the results were generated (some of the tools used do not provide information about which underlying model was applied).

Obtaining the appropriate data for an AGREE analysis using AI tools requires a precise, exact formulation of the task to be performed (i.e., prompting) as well as uploading a PDF or providing a link to the electronic version of the article. The exact prompt design (see Supporting Information) is crucial and includes defining subrequests into individual GAC rules (Table S1). It required a concrete action, such as selecting an answer from those suggested by the AGREE calculator, or providing a specific sample or waste quantity along with the required unit. An additional aspect was asking the chatbot to justify its choice, which allows for understanding the given answer and the underlying “thought process.”

Additionally, three experts (Experts 1, 2, and 3) were asked to extract the necessary data from the analyzed articles. These experts have extensive experience in developing new analytical methods. One of them has comprehensive experience in analytical chemistry and method evaluation (Expert 1), while the other two (Experts 2 and 3) are particularly involved in research on plant natural products.

## Results and Discussion

The research involved analyzing selected scientific articles (Articles 1–7) and extracting data helpful for environmental friendliness assessment (AGREE) using AI tools, while taking into account several aspects (Figure 1): testing five AI chatbots (ChatGPT, Copilot, Perplexity, Claude, and Gemini), testing different versions of the same AI tool (Perplexity and Perplexity Pro), evaluating the influence of the source file type (PDF or online article), and checking the reproducibility of results (Perplexity Pro). The articles were also evaluated by experienced researchers on the topics covered (Experts). The same list of prompts (see Table S1) was submitted to each of the AI tools to extract data from the seven representative articles^55–61^ (Table S2). After obtaining responses, all data were collected, critically analyzed, and the AGREE tool was used to assess the greenness of the analytical procedures described in the articles. Table S3 presents exemplary results, including the answers obtained, the AI-assigned weights, and the justification. This table includes a small excerpt of all the data obtained for selected articles (Articles 1, 3, and 7) and for selected GAC principles as an example.

It is worth noting that different AI tools assigned different weights to individual principles. The data in the articles were also interpreted differently, leading to the discrepancies in the responses obtained. For this reason, a justification that allows for understanding the reasoning behind each AI tool seems crucial. In the absence of clearly defined procedures, the analytical methods can be interpreted differently by both the chatbot and the experts, as observed. For example, for the data contained in Article 2, Rule 1, the AI tool responses were classified into three groups: off-line analysis; external sample pre- and treatment, and batch analysis (reduced number of steps); and external sample pre- and treatment and batch analysis (large number of steps). Generally, each of these responses is correct, depending on the interpretation. The situation is similar for experts, who, similarly to the AI, interpret differently, for example, the number of relevant steps in a given procedure. Some activities may be classified as necessary while others are not, resulting in different numbers of steps identified by both the AI and the experts. In the case of Rule 5 analysis of Article 3, the lack of miniaturization is clearly identified, but automation is interpreted differently. The use of autosamplers is often considered as an automated procedure, but when combined with manual sample preparation, it yields semiautomatic results. The last case in Table S3 (Rule 3 in Article 7) demonstrates excellent identification of the analytical equipment’s location relative to the test object (off-line in this case). The gas chromatograph was located in the laboratory, and both the samples and their preparation were performed separately. Practically all justifications support this interpretation.

It should be noted that discrepancies in data interpretation were observed for other rules as well, but they tended to follow similar relationships. For example, the minimum sample size was interpreted as the sample mass used for solution preparation and extraction (e.g., 50 mg), the volume after extraction (e.g., 1 mL), or the volume of the prepared sample injected into the chromatograph (e.g., 10 μL). The number of steps in the overall analytical procedure was also interpreted differently (actions such as mixing or shaking may not be considered as crucial as sonication or centrifugation). Sample volume can also be interpreted in the context of a single measurement or all replicates, which influences the reported values (e.g., 10 μL injection versus 30 μL). Similarly, for Article 1, experts provided three different answers regarding Rule 2: 0.5 g (amount used for extraction), 5 μL (single injection), and 0.015 mL (triplicate measurement). Derivatization, or its absence, was identified consistently in all cases. However, minor discrepancies occurred when determining operator safety by selecting from several options. Renewable sources, the most energy-intensive technique, the number of analytes determined in a single run, and sample throughput (samples analyzed per hour) were also identified in the same way in most cases, with minor discrepancies. The most significant discrepancy in the results occurred in the amount of waste generated. Wastes can be calculated for a single measurement or for all replicates. They may include or exclude mobile-phase consumption, the volume in which the sample was dissolved, or the volume used for the measurement. For example, for Article 7, the following responses were obtained for Rule 7: ∼5–10 mL, ∼1–2 mL, approximately 5 mL, approximately 5.1 mL, ∼10 mL. For ranges provided by AI (only few cases), the value most favorable to the method was consistently selected. This conservative approach did not influence final AGREE scores, which remained identical regardless of the specific value chosen within each range. The values for the amount of waste generated also varied among experts; in the extreme case corresponding to the analysis (in this article), the following responses were obtained: 10 mL, none, 0 mL, depending on whether the sample preparation process for the whole analytical procedure was taken into account.

Evaluating the repeated analyses performed by Perplexity Pro also revealed minor differences in the responses obtained, particularly for the number of analysis steps, the amount of waste, and the interpretation of the automation process. However, the overall trend was very similar.

Figure 2 presents the resulting pictograms from the AGREE analysis of selected scientific articles. For each article, pictograms are provided for the results extracted by each AI tool, the AI data’s repeatability, and the results based on expert assessment. Initial qualitative observation based on color shows high consistency between the final results (yellow for Article 2, orange for Article 3, and light green for Article 7).

**Figure 2:**
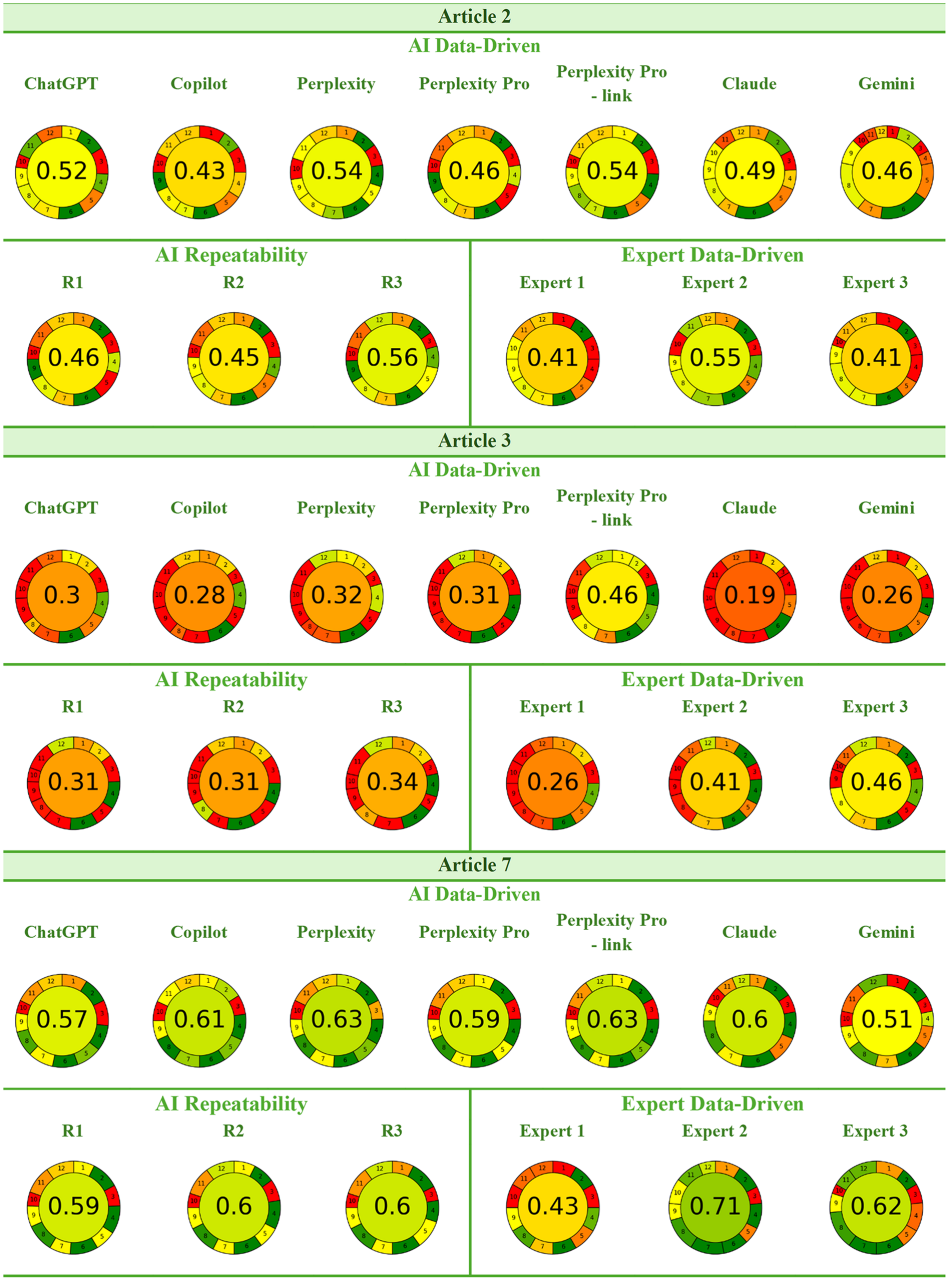
Exemplary AGREE pictograms for selected examples of the analyzed articles (R1-R3—repetitions).

Subsequent analysis of the obtained numerical results indicates good overall agreement. For example, for Article 2, the results fall within the following ranges: 0.43–0.54 for AI tools, 0.45–0.56 for AI repeatability, and 0.41–0.55 for experts, indicating good precision. Similar values were obtained for the remaining six articles. Differences in the observed results are shown by the different colors corresponding to the 12 GAC principles, which are presented around the main result in a circle. Deviations (e.g., observed as outliers in Figure 2) are natural and stem from several key factors, including the complexity of the scientific literature. Experts (despite their extensive knowledge and experience) differ in their interpretation of ambiguous data from scientific publications. Information can be presented in various formats (text, graphics, tables, or combinations of these formats) or as ranges for a given parameter. Each of these formats requires a distinct interpretation. Furthermore, scientific articles differ in how they present their data (detailed data or general descriptions), leading to natural discrepancies during data extraction (for both experts and AI). AI tools may interpret data differently (depending on the model), whereas experts are guided by broader scientific experience. The most important thing is that the observed differences do not change the overall score. All results follow a single, consistent trend. This confirms the robustness of both human and automated assessment. This consistency of results is far more important than minimal differences. Observed differences are a feature, not an error (all methods lead to a consistent conclusion about greenness).

For this reason, a more detailed analysis, considering each GAC principle separately (Figure 3), was conducted. In this figure, references were made to the selected three articles separately to maintain consistency with the previous figure’s pictograms. The analysis for each article shows the results obtained using all three previously mentioned methods (AI, AI repeatability, experts), this time broken down by the 12 GAC principles. Both visual analysis and the scores confirm high agreement in the interpretations between AI and the experts, with only minor deviations. The colors clearly illustrate where observed similarities and differences are. Generally, based on color analysis and the data overlapping, it is possible to isolate entire areas that contribute equally to the final score, e.g., Rules 3, 9, 10, and 11 in Article 3 marked in red, or Rules 2, 4, 6, and 8 in Article 7 marked in green. This trend was also observed for the remaining scientific publications.

**Figure 3:**
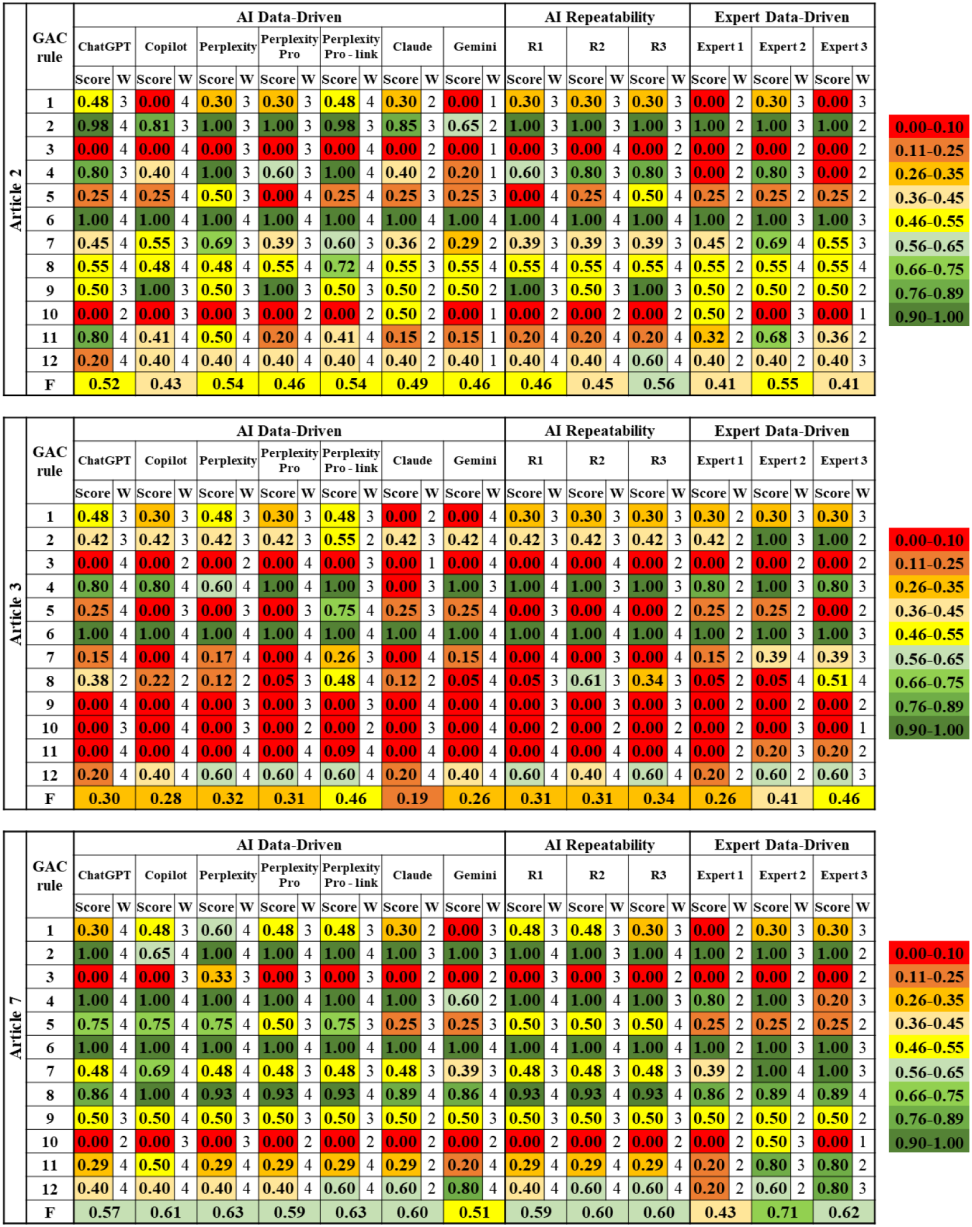
Exemplary detailed data analysis according to the 12 GAC rules for selected articles (F—final score, R1-R3—repetitions, W—weight).

Figure 4 (left side) shows the mean values for AI data-driven, AI repeatability, and expert data-driven analysis, further broken down by GAC principle. Qualitative analysis based on color coding and mean score values indicate a high degree of convergence, with only minor exceptions. However, the final result, taking into account the deviations, can be considered as the same. Similar observations with identical conclusions were made for the remaining four articles (data not shown). The AI repeatability standard deviation (equal to 0 in most cases) indicates very good precision.

**Figure 4:**
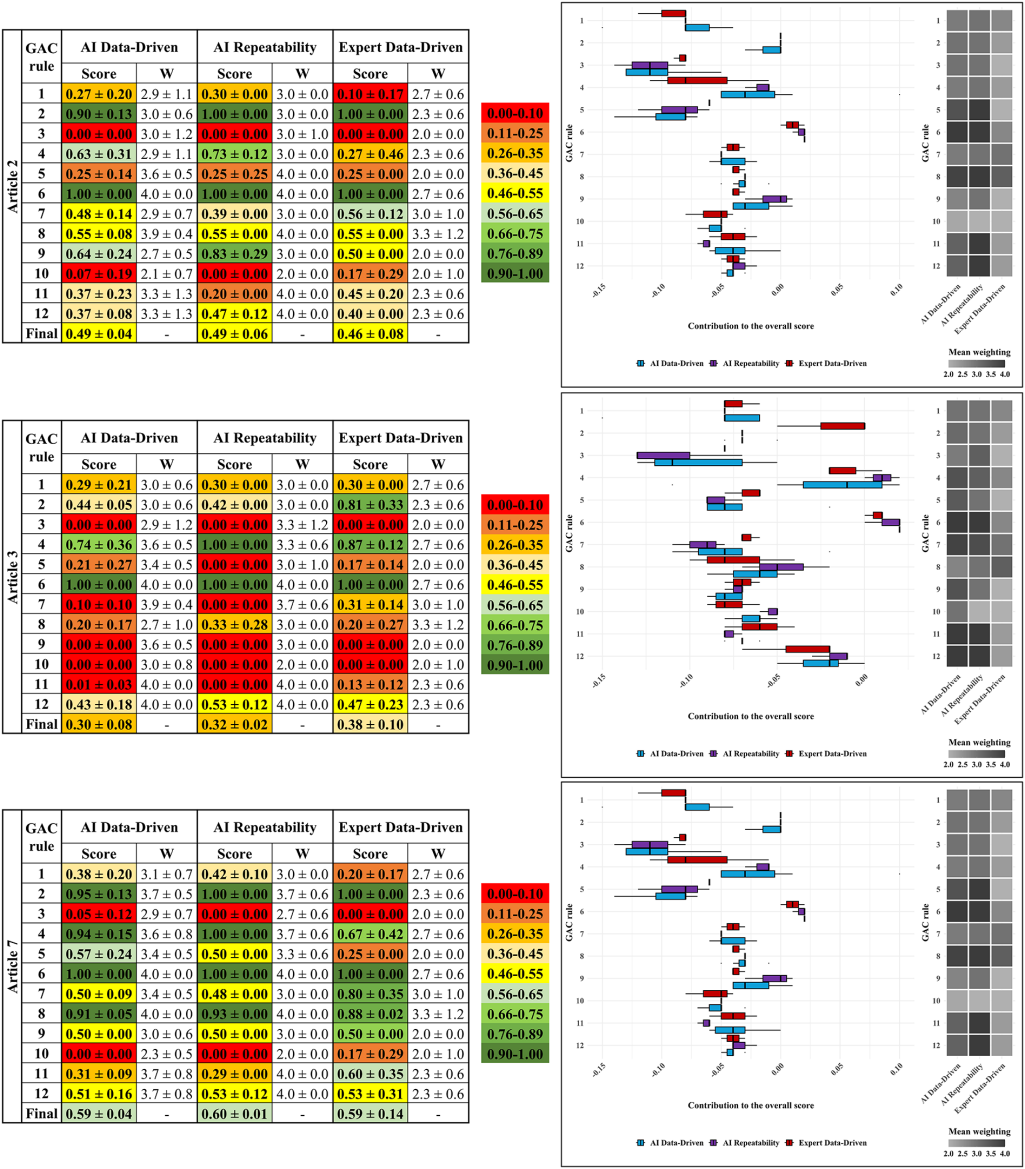
Exemplary general data analysis according to the 12 GAC rules for selected articles (left side: mean ± standard deviation (SD), right side: boxplots representing the contribution to the overall score and heatmaps the mean weighting) (F—final score, W—weight).

On the right side of this figure are graphs illustrating the contribution to the overall score for the 12 GAC principles. The close location of individual boxplots within the principles indicates a high degree of agreement between the three methods considered. Exceptions indicate rules where AI and experts differ in their interpretation of data from articles. Based on the position and size of the boxplots, it can be concluded that rules numbered 1 to 6 contribute the most to the final greenness score, with the greatest scatter observed. Wide boxplots indicate high variability in the impact of a specific rule, while narrow ones indicate a stable, well-defined contribution. The position relative to zero indicates an increasing or decreasing trend in the final score.

Rules with the highest weights contribute most to the final score. Based on Figure 4, specific rules (represented by red cells with high weights) can be identified where modifications to the analytical procedure and the reliability of AI tools should be assessed. If the differences between AI results and expert assessments are minor, AI can be considered a reliable tool to support the evaluation of the greenness of analytical methods.

A summary of the final results, along with the corresponding comparisons, for all seven analyzed articles is provided in Figure 5. The top table presents all final greenness scores obtained using AI tools, compared with AI repeatability and expert scores. Qualitative (based on colors) and numerical results-based analyses indicate the overall trend in evaluating environmental friendliness and which tools led to the outliers in a given case. For example, for Article 7, virtually all responses indicated the green nature of the analytical method, except for the results obtained with Gemini and Expert 1.

**Figure 5:**
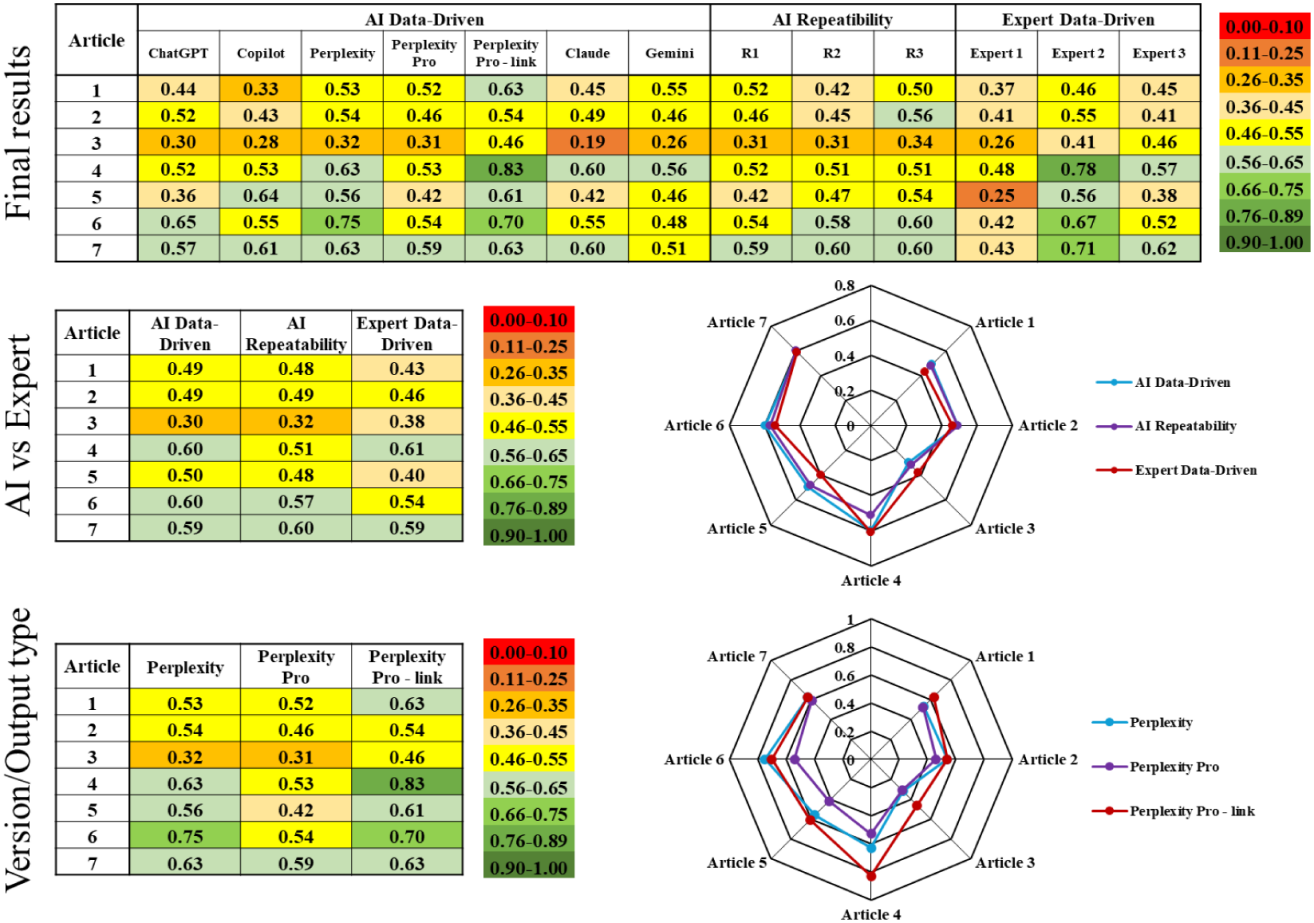
Final AGREE scores and comparison between AI and Experts, as well as between AI versions and output types, with corresponding radar plots (R1-R3—repetitions).

The radar plot in the middle of the figure was prepared to compare the average results between AI and experts. All results are consistent, which is particularly evident in Articles 2 and 7 (both visually and numerically). Additionally, based on the radar chart, similarities and differences are also clearly visible, and it is easy to identify which cases have the most significant differences (no overlap between points for Articles 3–5, but still marginal differences—ranging from 0.08 to 0.10 on the AGREE tool’s greenness scale). This demonstrates the high reliability of LLMs in assessing the greenness of analytical procedures compared to human experts.

The lower part of the figure, displaying a table and a radar chart, aims to compare the AI tool versions (standard and Pro) and the source file types (PDF or a link to the online version) when using Perplexity Pro. The Pro version provides extended access to Pro Search, advanced models, image/video generation, and higher usage limits (e.g., more queries per day, larger or more complex inputs). Analyzing both options yielded highly consistent results. The only minor differences occurred when using a link to the online version. The PDF and online versions of the article cannot be treated identically by AI models (due to differences in parsing, text fragmentation, and text encoding). In this case, the PDF file may provide AI tools with a more stable and complete text (despite parsing issues). The PDF file also lacks complete web navigation, which can generate noise and complicate the extraction of key data from the scientific text. In the online version, Supporting Information may be omitted, as they are available to experts. In subsequent tests, the source file will be combined with the online text.

After confirming the feasibility of using AI tools to extract data relevant to greenness assessment and establishing the high reliability of the results, it was decided to conduct a verification/comparison study. It involved selecting representative articles published in recent years using the AGREE tool, applying AI tools for data extraction, and comparing the results with those conducted by the authors. Five cases were selected that employed various analytical techniques in the area of plant natural products.^62–65^

Figure 6 presents the resulting pictograms for two selected cases (Cases 1 and 4, Table S2), comparing the use of different AI tools, AI repeatability, and the results obtained by the authors. For Case 1, the results differ significantly by approximately 20% in two situations: the AI application using a link to an online article and the authors’ assessment. The first situation is possible due to the different interpretation of the PDF file and the Website by the AI, as explained earlier. It is noteworthy that the authors’ result (0.94) is very close to the ideal method suggesting a potentially overly optimistic assessment. A similar relationship was detected in Case 2, which is based on the same article, employing the same analytical method but with a different application.

**Figure 6:**
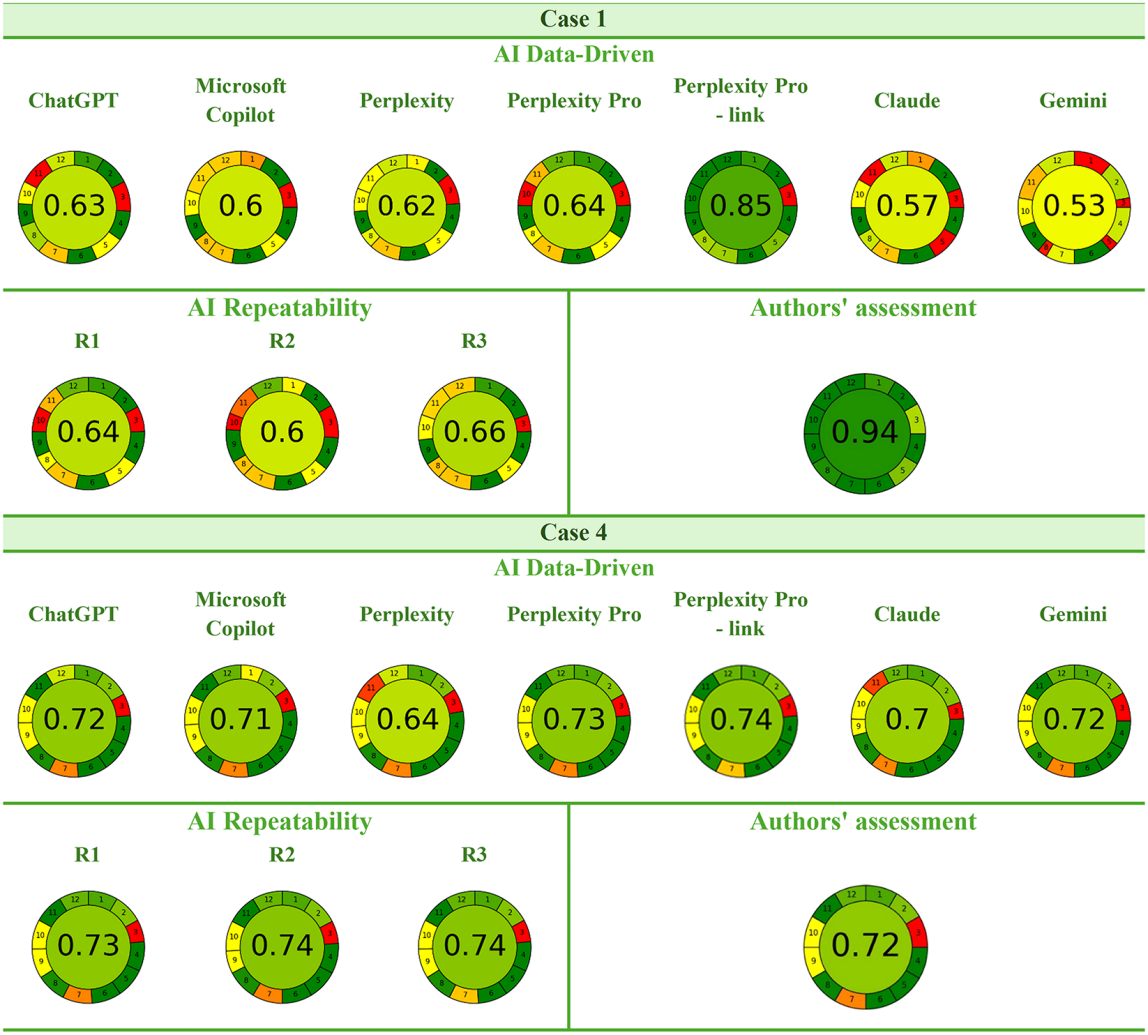
Exemplary AGREE pictograms for selected cases (R1-R3—repetitions).

The situation is different in Case 4, where the results from all AI tools (range 0.64–0.74) fluctuate around the value indicated by the authors (0.72). This is probably due to the detailed description of the analytical method which structurally corresponds to the principles of GAC. These observations allow for the use of AI tools to verify one’s own assessments of the developed procedures.

A summary of the final results for all five cases under consideration is presented in Figure 7. The table compares the results obtained based on AI data with the authors’ results. The authors’ results in Cases 1 and 2 differ significantly. In the remaining three cases, the results are more comparable. This table allows for a quick visual comparison based on colors and a comparison of the obtained scores. It is also possible to identify significantly different results, e.g., the result obtained using Copilot in Case 3 or using ChatGPT in Case 2. At this stage, it can also be noticed that Gemini lags slightly behind the other results. This involves giving significantly less importance to some principles, but this does not lead to drastically different conclusions.

**Figure 7:**
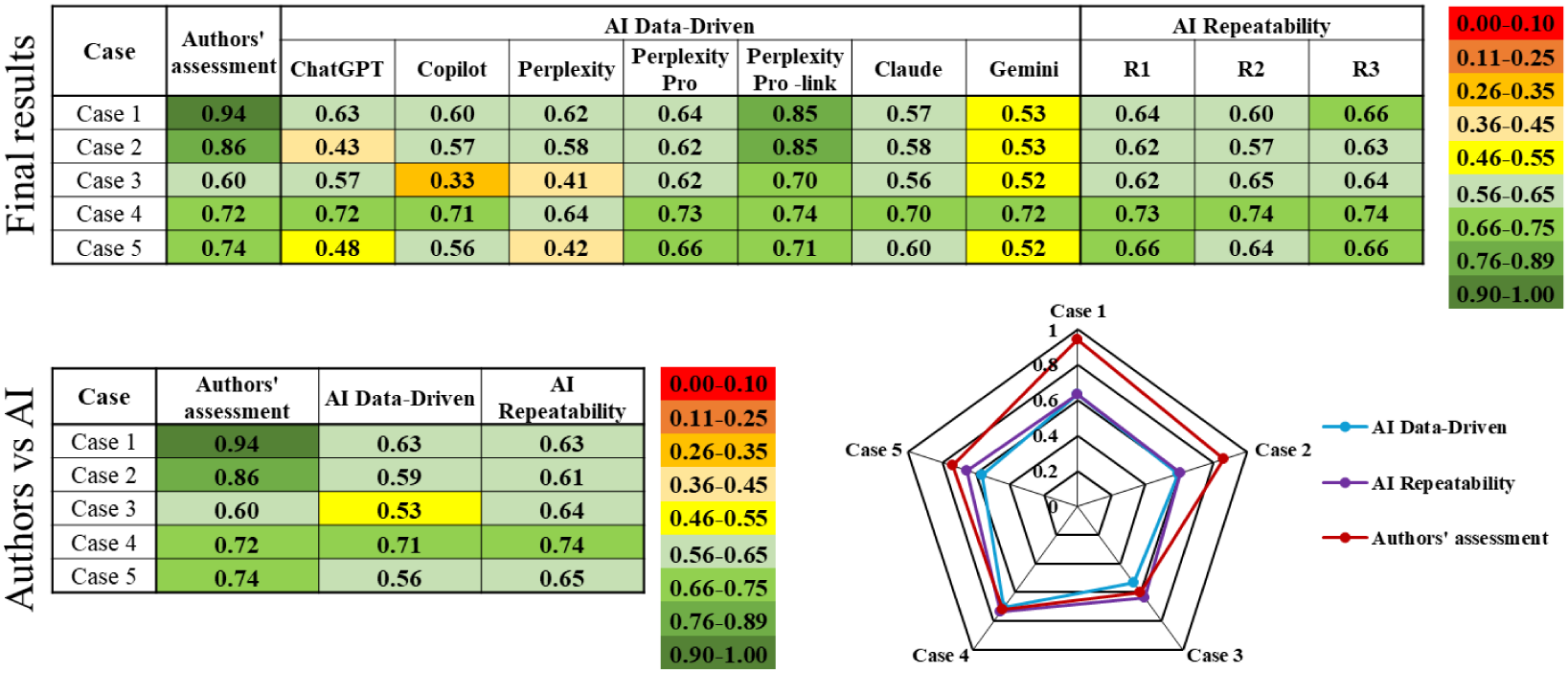
Final AGREE scores and comparison between Authors and AI, with corresponding radar plot (R1-R3—repetitions).

Comparing the mean values (bottom part of the figure: table and radar plot) allows to verify the authors’ evaluation. Acceptable agreement was found in Cases 3 and 5, while excellent agreement was confirmed in Case 4. The same dependencies were shown in radar plots, with particular deviations arising from overly optimistic assessments in Cases 1 and 2. In cases where the publication’s authors provided their own greenness assessment, this information might influence the LLMs’ responses, thereby (artificially) increasing consistency of scores. Results should be interpreted with caution, due to a potential carry-over of information into the model response.

In the final stage, four AI tools (Elicit—a tool for literature review, searching for publications and automatic extraction of data from scientific articles; Paperguide—used for literature search, working with PDF files, creating summaries, managing citations and supporting the writing of scientific texts; SciSpace—used for literature search, interactive reading of PDF files, information synthesis and supporting work on publications; and Consensus—a tool for finding answers based on scientific publications, useful for research questions and quick assessment of a given thesis) dedicated to scientific research were used to compare the AGREE scores. Figure 8 presents the results obtained with these tools compared to the other methods of assessing greenness used in the article.

**Figure 8:**
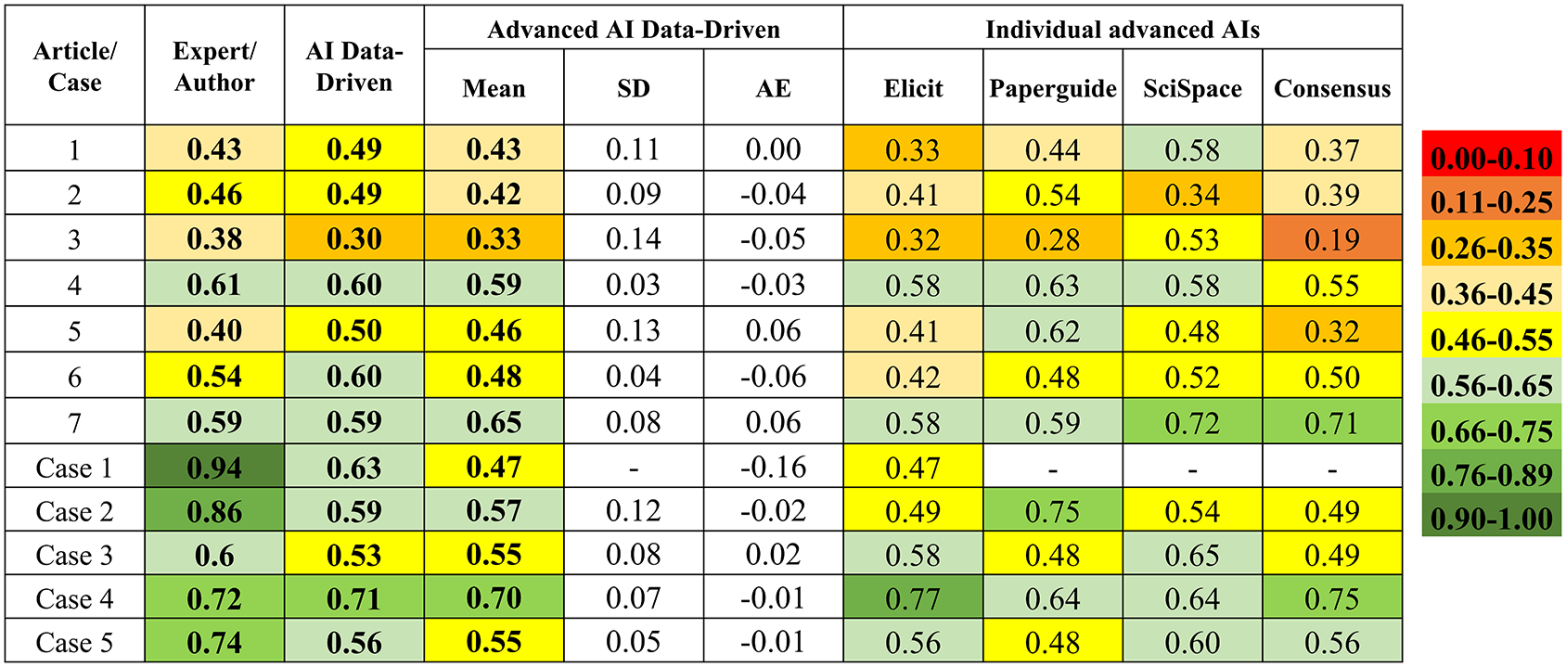
Summary of results obtained using advanced AI tools (SD—standard deviation, AE—absolute error). AE was calculated for seven articles against expert analysis, and for selected cases against basic AI tools (values marked with *)—such interpretation serves as the more reliable reference.

A qualitative comparison of the results obtained for the seven scientific articles (based on assigned colors) and a quantitative comparison based on their values indicates high agreement with both expert assessment and basic AI tools. The maximum absolute error (AE) value is 0.06, indicating high agreement in the evaluation process of the analytical methods described in these articles. Additionally, both negative and positive values are observed, indicating underestimation or overestimation. However, they oscillate around the exact value. Selected cases for verification were also included in this comparison. It is worth noting that there is high agreement between the basic and advanced AI tools (AE ≤ 0.02), whereas in most cases, the values assessed by the authors differ significantly. An interesting situation was observed in Cases 1 and 2, which are based on the same article but refer to a pure compound and a biological sample, respectively. In these cases, after applying the same prompt, the advanced AI tools focused primarily on the biological sample data, thus preventing the calculation of scores for Paperguide, SciSpace, and Consensus. The observed discrepancies in the four AGREE scores for Elicit, Paperguide, SciSpace, and Consensus are described by SD values (not exceeding 0.14, with the exception of Case 1, which had incomplete data representation), which are comparable across both experts and general AI tools and indicate high stability of the results. These values may be related to how data are presented in scientific articles, indicating sensitivity to input parameters. For this reason, it is recommended to use at least three different AI tools (basic and/or advanced) and calculate the average. The more such tools are used during the assessment and the more repetitions are performed, the more reliable the value will be. Low SD and AE values (close to 0) indicate high consistency and repeatability across AI tools, demonstrating that different models converge on similar interpretations.

It is important to note that each tool used in the experiments (ChatGPT, Copilot, Perplexity, Claude, and Gemini) follows different data-processing principles, leading to differences in handling PDF files, graphical objects, and citations. ChatGPT performs fast, accurate data extraction from text, but can replace missing data with nonexistent values. Problems may also arise during scan analysis. Copilot handles scanned files well through Optical Character Recognition (OCR), integrates with Microsoft Office, and is well-suited for summarizing and presenting data in tabular form, as well as working with tables. Perplexity is well-suited for scientific research due to its appropriate indexing and retrieval-based search for relevant text fragments. However, it may be limited by its inability to load entire PDF files (40 MB per file). Claude is characterized by high accuracy in analyzing PDF files and by the absence of data hallucinations, thanks to JavaScript Object Notation (JSON)—a scheme for precise responses. Furthermore, it has built-in OCR, enabling visual analysis of images and tables. Similar to Claude, Gemini handles precise responses (via JSON) from text and visualizations, and enables table extraction. These features yield different responses when extracting data from scientific articles.

The limited reproducibility of results from AI tools (both basic and advanced) is natural and explainable by the fact that generative models exhibit inherent variability in their responses.^66,67^ This variability depends on the prompt formulation, input format, and data interpretation. Consequently, even for the same article and task, the results may differ across iterations, which can affect the final AGREE score. Such variability does not disqualify AI models from being useful. However, it indicates the need to treat them as supporting tools (at this stage, they do not replace expert assessment, especially in ambiguous cases) that can effectively support a quick literature review and preliminary greenness assessment.

LLMs can support a more standardized, objective assessment of criteria in the AGREE tool (due to the use of the same prompt and a consistent data extraction scheme). Their use reduces the subjectivity that can be associated with expert work, especially with the large number of methods being compared and their similarity. However, this does not eliminate subjectivity—LLM results still depend on the description used in the article, the unambiguity of the wording, and the form of information.

Using these tools also results in certain limitations in data extraction from scientific articles. One such limitation is the potential to generate false/nonexistent data, such as the amount of waste generated or its toxicity assessment, which can significantly distort the greenness assessment. This type of false data is challenging to detect without a detailed description of the analytical procedure. In our analyses, there were a few instances in which the AI tool calculated energy consumption based on literature data or “miscalculated” the amount of toxic reagents. Inaccuracies were also noticed when using a link rather than a PDF. Current chatbots lack specialized scientific terminology, for example, regarding GAC, which can lead to erroneous interpretation of various laboratory procedures. Data presented in visual form, such as charts, drawings, or pictograms, which are often included in scientific articles, may not be detected by AI, potentially leading to the loss of important information. Analyzing large numbers of articles is also limited due to computational efficiency. Legal and ethical considerations also pose a limitation, as the analyses presented here are only possible for articles published in open-access journals. Testing on unpublished manuscripts or articles with restricted access may violate university and data protection policies. Data and descriptions can be treated as a training element for AI tools, which poses a risk, especially for unpublished research. It is also important to note that this article only presents a snapshot from October-November 2025, and May 2026. Companies’ AI models are constantly evolving and improving, which could significantly impact their capabilities in the coming months. The research is burdened by the fact that AI models are continually updated, which limits the ability to replicate the exact model versions, and the published results will not remain fully up to date. This limits strict repeatability but reflects real-world usage conditions, where users employ the most current and best-performing models available. However, analyses conducted demonstrate a universal strategy for assessing the greenness of analytical methods.

Despite the aforementioned limitations, using AI tools as a verification/comparison process is highly beneficial, since it confirms the results of one’s own evaluation and can help to identify errors. The use of several advanced AI tools is a good solution to verify the obtained greenness scores. Furthermore, it can be used for rapid, automated data extraction. However, it is important to remember that to obtain reliable data, a well-defined prompt must be prepared, preferably with justification. We recommend using several AI tools (at least three) and calculating an average value. Alternatively, the assessment can be repeated several times using the same tool (at least three repetitions). This average result can be compared with the user’s own assessment. The analytical procedure should be clearly and concisely described in the experimental section. Depending on the tool used to evaluate the method, this description can be adapted to the specific criteria being assessed. The description should also specify what it refers to, e.g., 1 sample, 1 measurement, the number of repetitions for a given sample, and whether certain laboratory activities can be performed simultaneously. A well-described, as unambiguous as possible, analytical procedure will enable AI tools to extract data faster, thereby improving the reliability of the greenness assessment while avoiding unnecessary overinterpretation.

The sustainable nature of scientific research in general requires special attention in evaluating the greenness of analytical methods. The proposed AI data extraction method for greenness not only enables method evaluation but also leads to systemic monitoring of the environmental impact of analytical laboratories. It can be a source of better planning and the implementation of general management policies, which will translate into better resource allocation (consumables, chemical reagents, energy, and equipment wear) and reduced chemical pollution. By appropriately extracting data, it is possible to identify sustainable waste management solutions and quickly update best practices. The proposed approach reduces time and costs, facilitates the planning of such activities, and reduces resource consumption. The use of open-access tools and automation provides access to the same knowledge about green methods regardless of individual institutions’ financial resources, reducing disparities between rich and poor institutions. Open data, as well as transparent and repeatable assessments, support the transformation toward fair and responsible chemical analytics (SDG 12). The presented analyses of scientific articles can be extended to standards, procedures, and other documents, constituting an entirely new type of knowledge, leading to the creation of appropriate databases. Sustainable practices can be implemented in various analytical and scientific laboratories, legal regulations, certification systems, and supply chains, which already represent a significant economic and systemic dimension.

## Conclusions

The analyses conducted confirmed the feasibility of using AI tools for automatic data extraction from scientific publications relevant for assessing the greenness of the described analytical method in a sustainable framework. High accuracy was demonstrated in the general and detailed AGREE analyses using AI-derived data, compared with expert assessments. The research verified the usefulness of various freely available chatbots; the results were comparable across the five most popular tools. Greater convergence of results was achieved with a PDF of the scientific article rather than with a link to the published online version. There were no significant differences in results when comparing the traditional (free) version with the Pro version, which allows for more analyses without limitations. However, obtaining specific data is only possible with adequate prompt design, preferably with a justification, which enables verification and comparison of the extracted data. AI tools allow subjective verification of previously performed greenness assessments. Advanced AI tools demonstrated high effectiveness in automated data extraction for manual greenness assessment using the AGREE tool, achieving results consistent with expert assessments. This confirms that their use can significantly support and streamline the analytical method evaluation processes while maintaining good repeatability and reliability. Currently, LLMs alone cannot ensure sufficient data quality and reliability. Therefore, their results should be considered a supplement to expert analysis. Using multiple AI tools and averaging results can improve evaluation accuracy—but expert verification and interpretation are still recommended. It is possible to extend this application to other method evaluation tools, particularly White Analytical Chemistry. It is recommended to include a detailed description of the procedures performed in the experimental section, using a scheme similar to the 12 GAC rules, and to indicate the data reference (single measurement, replicates, single sample, simultaneous activities). The use of AI tools can improve the sustainability of laboratory practices by assisting in the assessment of procedures as well as in the prioritization of methods with low environmental impact or reduced costs. This supports the transformation of analytical practices, avoiding the production of waste and the depletion of valuable resources, and consequently leads to intergenerational equity in resource-constrained environments. Integrating AI-based analytical method evaluation with databases and open repositories in the future, could facilitate the transition toward a sustainable development in chemistry. Future developments may include API-based automation linking AI-driven data extraction with greenness assessment tools like AGREE.

## Supplementary Material



## Data Availability

Data available upon request from the authors.

## Notes

Table S1: prompt design for reliable data extraction from scientific publications to calculate AGREE scores; Table S2: summary of articles selected for analysis and verification/comparison; Table S3: exemplary answers and justifications from AI tools, where W—weight (PDF)
