# Supplementary material for: AI-Driven Data Extraction and AGREE-Based Greenness Evaluation of Analytical Methods for Natural Products: Insights from Selected Studies
Source: Anal Chem. 2026 Jul 29;98(31):22595–608. doi: 10.1021/acs.analchem.6c02529 (PMC13470974; doi:10.1021/acs.analchem.6c02529)
Supplement: Supplementary file 1 [file ac6c02529_si_001.pdf]

Supporting Information

AI-Driven Data Extraction and AGREE-Based Greenness Evaluation of Analytical Methods for Natural Products: Insights from Selected Studies

Paweł Świt*[a,b], Fabian Hammerle*[b], Markus Ganzera[b]

[a] Institute of Chemistry, Faculty of Science and Technology, University of Silesia in Katowice, Szkolna 9, 40-006 Katowice, Poland

[b] Institute of Pharmacy, Department of Pharmacognosy, University of Innsbruck, Innrain 80-82, 6020 Innsbruck, Austria

* Corresponding author: fabian.hammerle@uibk.ac.at, pawel.swit@us.edu.pl

Table of contents

**Table S1**. Prompt design for reliable data extraction from scientific publications to calculate AGREE scores.	S2-S3
**Table S2.** Summary of articles selected for analysis and verification/comparison.	S4-S6
**Table S3.** Exemplary answers and justifications from AI tools, where: W - weight.	S7-S10

Table S1. Prompt design for reliable data extraction from scientific publications to calculate AGREE scores.

I kindly ask you to analyze the article file and complete the following 14 points, strictly following the recommendations.<br><br>**1. Direct analytical techniques should be applied to avoid sample treatment.**<br><br>Please select the best statement from the list (available options are separated by the ";" sign): remote sensing without sample damage; remote sensing with little physical damage; non-invasive analysis; in-field sampling and direct analysis; in-field sampling and on-line analysis; on-line analysis; at-line analysis; off-line analysis; external sample pre- and treatment and batch analysis (reduced number of steps); external sample pre- and treatment and batch analysis (large number of steps).<br>Rate the importance of this feature on a scale of 1-4 (1 - least, 4 - most).<br>Provide a justification for selecting a statement from the list.<br><br>**2. Minimal sample size and minimal number of samples are goals.**<br>Enter the amount of sample in either g or mL.<br>Rate the importance of this feature on a scale of 1-4 (1 - least, 4 - most).<br>Provide a justification for your choice of sample amount.<br><br>**3. In Situ Measurements Should Be Performed. What is positioning of the analytical device?**<br>Please select the best statement from the list (available options are separated by the ";" sign): off-line; at-line; on-line; in-line.<br>Rate the importance of this feature on a scale of 1-4 (1 - least, 4 - most).<br>Provide a justification for selecting a statement from the list.<br><br>4. **Integration of analytical processes and operations saves energy and reduces the use of reagents - selection from: How many major, distinct steps are there in the sample preparation procedure? These include e.g., sonication, mineralization, centrifugation, derivatization, extraction, etc.**<br>Please select the best statement from the list (available options are separated by the ";" sign): 3 or fewer; 4; 5; 6; 7; 8 or more.<br>Rate the importance of this feature on a scale of 1-4 (1 - least, 4 - most).<br>Provide a justification for selecting a statement from the list.<br><br>**5. Automated and miniaturized methods should be selected. Degree of automation.**<br>Please select the best statement from the list (available options are separated by the ";" sign): automatic; semi-automatic; manual.<br>Rate the importance of this feature on a scale of 1-4 (1 - least, 4 - most).<br>Provide a justification for selecting a statement from the list.<br><br>**6. Automated and miniaturized methods should be selected. Sample preparation.**<br>Please select the best statement from the list (available options are separated by the ";" sign): none or miniaturized; not miniaturized.<br>Rate the importance of this feature on a scale of 1-4 (1 - least, 4 - most). Should be the same like for Point 5 – Degree of automation.<br>Provide a justification for selecting a statement from the list.<br><br>**7. Derivatization should be avoided.**<br>Select derivatization agents (if used) and CAS number.<br>Rate the importance of this feature on a scale of 1-4 (1 - least, 4 - most).<br>Provide a justification for your choice of derivatization agents.<br><br>**8. Generation of larger volume of analytical waste should be avoided, and proper management of analytical waste should be provided.**<br>Enter the amount of waste in g or mL.

Rate the importance of this feature on a scale of 1-4 (1 - least, 4 - most).
Provide a justification for the selection of waste quantities.

9. Multi-analyte or multi-parameter methods are preferred versus methods using one analyte at a time.
Enter the number of analytes determined in a single run.
Rate the importance of this feature on a scale of 1-4 (1 - least, 4 - most).
Provide a justification for the selection of the number of analytes determined in a single run.

10. Multi-analyte or multi-parameter methods are preferred versus methods using one analyte at a time.
Enter the sample throughput (samples analysed per hour).
Rate the importance of this feature on a scale of 1-4 (1 - least, 4 - most). Should be the same like for Point 9.
Provide a justification for your choice of sample throughput (samples analyzed per hour).

11. The use of energy should be minimized.
Select the most energy-intensive technique used in the method (available options are separated by the ";" sign): None; FTIR; Hot plate solvent evaporation (<10 min); Rotary evaporation; Needle evaporation; Ultrasound-assisted extraction; SPE and SPME; Microbiological assays; Immunoassay; Spectrofluorymetry; Titration; UPLC; UV-Vis Spectrometry; Energy dispersive X-ray fluorescence; Potentiometry; Non-instrumental detection; Hot plate solvent evaporation (10-150 min); Accelerated solvent extraction; Supercritical fluid extraction; Microwave assisted extraction; Flame atomic absorption spectrometry; Electrothermal atomic absorption spectrometry; GC; ICP MS; ICP-OES; LC; Hot plate solvent evaporation (> 150 min); Soxhlet extraction; NMR; GC-MS; LC-MS; X-ray diffractometry.
Alternatively, estimate the total power consumption of a single analysis in kWh.
Rate the importance of this feature on a scale of 1-4 (1 - least, 4 - most).
Provide a justification for your selection from the list or for estimating the total power consumption of a single analysis in kWh.

12. Reagents obtained from renewable sources should be preferred.
Select the type of reagents (available options are separated by the ";" sign): No reagents; All regents are bio-based; Some reagents are bio-based; None of the reagents are from bio-based sources.
Rate the importance of this feature on a scale of 1-4 (1 - least, 4 - most).
Provide a justification for selecting a statement from the list.

13. Toxic reagents should be eliminated or replaced.
Does the method involve the use of toxic reagents or solvents? Please select the best statement from the list (available options are separated by the ";" sign): No; Yes.
Estimate the amount of toxic reagents or solvents in g or mL.
Rate the importance of this feature on a scale of 1-4 (1 - least, 4 - most).
Provide a justification for selecting a statement from the list.

14. Operator's safety should be increased.
Select the threats which are not avoided (can be more than 1) (available options are separated by the ";" sign): toxic to aquatic life; bioacumulative; persistent; highly flammable; highly oxidizable; explosive; corrosive.
Rate the importance of this feature on a scale of 1-4 (1 - least, 4 - most).
Provide a justification for selecting a statement from the list.

Table S2. Summary of articles selected for analysis and verification/comparison.

Article/Case	Bibliographic data	Short description
Article 1	Fernando L. Vera-Baquero, Natalia Casado, Sonia Morante-Zarcero, Isabel Sierra, Improving the food safety of bakery products by simultaneously monitoring the occurrence of pyrrolizidine, tropane and opium alkaloids, Food Chemistry 460 (2024) 140769, https://doi.org/10.1016/j.foodchem.2024.140769.	This article describes the development of a new analytical method for the extraction and determination of plant-derived alkaloids (pyrrolizidine, tropane, and opium alkaloids) in samples of commercially available bakery products. A method based on the application of liquid chromatography with mass spectrometry was developed, optimized, and validated. At least one alkaloid was detected and identified in 15 analyzed samples. The aim of the study was to ensure food safety and compliance with actual regulations. The need for simultaneous monitoring of these compounds in food was also emphasized, and the inclusion of opium alkaloids in current regulations was suggested.
Article 2	Juan Pablo Yasmo Perez, Carolina Chegwin Angarita, Omar David Saldarriaga Ochoa, Sergio Andrés Urrego Restrepo, Development and validation of a HPLC-DAD method for determining the content of tryptamines in methanolic extracts of fruiting bodies of mushrooms belonging to species of the Psilocybe genus, Talanta 290 (2025) 127777, https://doi.org/10.1016/j.talanta.2025.127777.	This work presents the development and validation of analytical methods using high-performance liquid chromatography with diode array detection to determine tryptamine content in *Psilocybe* mushroom fruiting body samples (sold in Colombia). The developed method demonstrated satisfactory validation parameters. This method analyzed tryptamines in 19 mushroom samples from the Medellin area (tryptamine content within the sample range 0.01%–0.73%).
Article 3	Poonam Agrawal, Kirti Laddha, Development of validated high-performance thin layer chromatography for quantification of aristolochic acid in different species of the Aristolochiaceae family, Journal of Food and Drug Analysis 25 (2017) 425-429, https://doi.org/10.1016/j.jfda.2016.06.006.	This paper presents the development of a chromatographic method for the isolation (solvent extraction, precipitation, recrystallization) and determination of aristolochic acid in two plant samples (*Aristolochia indica* stem, *Apama siliquosa* root). An analytical method based on using high-performance thin-layer chromatography with separation on silica gel plates was developed and validated. The approach proved to be effective in analyzing herbal materials and diverse species of the Aristolochiaceae family.
Article 4	Zhao-Yan Ren, Yu Zhang, Yan-Ping Shi, Simultaneous determination of nine flavonoids in Anaphalis margaritacea by capillary zone electrophoresis, Talanta 78 (2009) 959-963, https://doi.org/10.1016/j.talanta.2009.01.006.	This article describes the development of a method based on capillary zone electrophoresis for the determination of nine flavonoids in Tibetan medicine - *Anaphalis margaritacea*. The developed method is characterized by good accuracy, satisfactory repeatability, a short

		analysis time, and high separation efficiency, which makes it particularly useful for quality control.
Article 5	Punam Thapa, Hye Mi Kim, Joon-Pyo Hong, Ranhee Kim, Sunil Babu Paudel, Hyukjae Choi, Dae Sik Jang, Joo-Won Nam, Absolute Quantification of Isoflavones in the Flowers of Pueraria lobata by qHNMR, Plants 11 (2022) 548, https://doi.org/10.3390/plants11040548.	This paper presents the development of a new method using quantitative proton nuclear magnetic resonance spectroscopy and an internal standard for the determination of seven isoflavones in Puerariae Flos (the flower of *Pueraria lobata* - a medicinal plant native to Korea, China, and Japan). The developed method was characterized by simplicity, good sensitivity, and reliable results, which were compared to the reactive method (liquid chromatography with UV detection). The approach is based on proton signals from H-2 singlets of C-ring in the range of 8.2–8.5 ppm (signal separation, no signal overlap).
Article 6	Michael Zwerger, Markus Ganzera, Analysis of boswellic acids in dietary supplements containing Indian frankincense (Boswellia serrata) by Supercritical Fluid Chromatography, Journal of Pharmaceutical and Biomedical Analysis 201 (2021) 114106, https://doi.org/10.1016/j.jpba.2021.114106.	This article describes the first application of supercritical fluid chromatography to determine six boswellic acids in *Boswellia serrata* extracts in a short time (<6 min) and with high selectivity. The developed approach met the validation criteria to a high degree and was successfully tested on several natural products (dietary supplements) containing mainly β-boswellic acid.
Article 7	Waseem Gul, Iram Shahzadi, Nandakumara Sarma, Nam-Cheol Kim, Mahmoud A. ElSohly, Development and Validation of a GC-FID Method for the Quantitation of Δ 8-Tetrahydrocannabinol and Impurities Found in Synthetic Δ 8-Tetrahydrocannabinol and Vaping Products, Planta Medica 90 (2024) 316-332, https://doi.org/10.1055/a-2249-7824.	This work involves the development and validation of a simple and rapid method using gas chromatography with flame ionization detection for the determination of delta-8-tetrahydrocannabinol and its contaminants. This method was used to test 21 commercially available vaporization products, demonstrating exceedances of the tested contaminant limits. This approach could be an important tool for monitoring contamination with these substances in commercially available products.
Cases 1 and 2	Heba Abd El-Aziz, Nada E. Hammouda, Fathallah Belal, Heba Samir Elama, Eco-Friendly Analytical Approach for Sensitive Spectrofluorimetric Determination of the Flavonoid Chrysin in Capsules and Human Plasma, Journal of Fluorescence 35 (2025) 6399-6408, https://doi.org/10.1007/s10895-024-03962-9.	This article presents the development of a new spectrofluorometric method for the determination of chrysin (a plant flavonoid with therapeutic activity) utilizing the compound's natural fluorescence. The study analyzed the pure form and tablet samples (commercial chrysin capsules) (**Case 1**) and human plasma enriched with the investigational substance (**Case 2**). The method demonstrated high analytical reliability

		and environmental friendliness due to the use of distilled water as a solvent. Furthermore, it was sensitive, rapid, and reliable.
Case 3	Pornpoom Angsuwattana, Wisut Wichitnithad, Yanyong Punpreuk, Hasseri Halim, Boonchoo Sritularak, Worathat Thitikornpong, Pornchai Rojsitthisak, Development of a Simplified Heart-Cutting Two-Dimensional Liquid Chromatography Method for Green Analysis of Four Polyphenols in Aerides Plants, ACS Sustainable Chemistry & Engineering 13 (2025) 4489-4498, https://doi.org/10.1021/acssuschemeng.4c10145.	This paper describes the development of a new strategy using two-dimensional liquid chromatography for the simultaneous determination of four polyphenols (acting as analytical markers) in plants of the *Aerides* genus, native to Southeast Asia. Excellent peak shapes and resolution were achieved, along with satisfactory validation parameters, providing a basis for quality control and standardization of the tested products. Furthermore, the method demonstrated high sensitivity and selectivity, as well as reduced matrix interference. The advantage of the developed method - the reduction of negative environmental impact - was also emphasized.
Case 4	Daniel Andrade Rabelo Lima, Eliete Costa Alves, Jeancarlo Pereira dos Anjos, Greener method proposal for the determination of phenolic compounds and coumarins in aged sugarcane spirits using green bio-based solvents in HPLC-DAD-FLD system, Green Analytical Chemistry 10 (2024) 100138, https://doi.org/10.1016/j.greeac.2024.100138.	This paper describes the development of an eco-friendly method using high-performance liquid chromatography with diode array and fluorescence detection to determine phenolic compounds and coumarins in samples of sugarcane spirit aged in barrels made of various types of wood. The primary objective was to propose an eco-friendly method utilizing organic biological solvents (ethanol and acetic acid), reducing analysis time and minimizing waste, which aligns with GAC principles.
Case 5	Eleonora Oliva, Aina Mir-Cerda, Manuel Sergi, Merce Granados, Sonia Sentellas, Javier Saurina, Green extraction of phenolic compounds from strawberry waste based on natural deep eutectic solvents, International Journal of Food Science & Technology 59 (2024) 3967-3977, https://doi.org/10.1111/ijfs.17148.	This article compares the extraction efficiency of phenolic compounds using traditional organic solvents and environmentally friendly Natural Deep Eutectic Solvents from strawberry residues, as an example of agro-food byproducts. During the optimization phase, various combinations of these reagents were tested, and the samples were analyzed using liquid chromatography-mass spectrometry. It was demonstrated that alternative solvents are characterized by high extraction efficiency, are rapidly and straightforwardly applied, are environmentally friendly, and can replace traditional ones.

Table S3. Exemplary answers and justifications from AI tools, where: W - weight.

AI tools	Article 2 - Rule 1		Article 3 - Rule 5		Article 7 - Rule 3	
	**Answer**	**Justification**	**Answer**	**Justification**	**Answer**	**Justification**
ChatGPT	off-line analysis (W=3)	*The method requires extracting dried fruiting bodies with methanol (ratio 1:3 mL/g), four ultrasound-assisted cycles, rotary evaporation and reconstitution prior to injection — i.e. the analysis is performed off-line after laboratory sample preparation rather than non-invasively or in-field. This is explicitly described in the Methods (extraction, filtering, rotary evaporator, reconstitution).*	semi-automatic (W=4)	*The method uses instrumental supports (Desaga applicator, automated TLC scanner/densitometer) for application and detection, but sample preparation (Soxhlet extraction, concentration, recrystallization, volumetric adjustments) is manual or conventional laboratory-scale — therefore semi-automatic is the best fit. Semi-automation improves repeatability (they report good RSDs) but the sample-prep remains labor- and time-intensive, so importance is near-top.*	off-line (W=4)	*All preparation is done off-line (weighing, dissolution, sonication, volumetric dilution) and measurement is performed in the laboratory on an Agilent 6890 GC-FID instrument (not at-line/on-line/in-line). The instrument and autosampler are lab-based.*
			not miniaturized (W=4)	*Sample prep uses classical Soxhlet extraction (50 mL methanol for 5 g sample, 5 h), concentration, and recrystallization for isolation — none of which are miniaturized microextraction approaches. Because the method relies on conventional-volume Soxhlet and evaporation, it is not miniaturized; importance is high because miniaturization would reduce solvent use and hazard.*		
Copilot	external sample pre-	*The method involves multiple steps including extraction, filtration,*	manual (W=3)	*Sample preparation and HPTLC spotting are manually performed.*	off-line (W=3)	*The GC-FID setup is located in a laboratory and does not allow for in*

	and treatment and batch analysis (large number of steps) (W=4)	*drying, reconstitution, and chromatographic separation. These are not direct or in-field techniques, but rather extensive lab-based procedures.*		*While precise, automation could improve throughput and reproducibility.*		*situ or real-time analysis at the point of sample generation.*
			not miniaturized (W=3)	*The method uses conventional Soxhlet extraction and volumetric flasks, indicating standard-scale preparation rather than miniaturized formats.*		
Perplexity	external sample pre- and treatment and batch analysis (reduced number of steps) (W=3)	*The developed method employs external extraction using methanol and subsequent filtration, drying, and dissolution before HPLC-DAD analysis. While these are pre-treatment steps, the procedure avoids unnecessary complexity; however, it does not qualify as direct, in-field, or online analysis due to the need for extraction and preparation steps. The reduced number of steps compared to more exhaustive batch methods justifies this choice.*	manual (W=3)	*The procedures—extraction, recrystallization, spotting on HPTLC plates, and densitometric scanning—are carried out manually. Increased automation would enhance throughput, reproducibility, and operator safety.*	at-line (W=3)	*The analytical method is conducted rapidly in a lab following minimal preparation, suitable as at-line analysis rather than on-line or in-line settings.*
			not miniaturized (W=3)	*Extraction, concentration, and application are manual and not miniaturized, involving standard lab-scale volumes and glassware. Miniaturization could lower solvent/reagent use and environmental impact but is not implemented here.*		
Perplexity Pro	off-line analysis (external sample pre- and treatment and batch analysis with reduced number of steps) (W=3)	*The method developed involves sample extraction using methanol with ultrasound-assisted extraction and rotary evaporation prior to HPLC-DAD analysis, which constitutes off-line batch analysis with sample pre-treatment steps. The method aims to be easily implemented and replicable but does*	manual (W=3)	*The extraction, recrystallization, and HPTLC spotting are all manual processes.*	off-line (W=3)	*The sample is prepared off-line prior to GC-FID analysis; no real-time or in-line measurement setup is used.*
			not miniaturized (W=3)	*Sample preparation uses conventional solvent extraction and recrystallization at gram scale, no miniaturization or automation applied.*		

		not use in-field or on-line direct analysis techniques.				
Perplexity Pro - link	off-line analysis (W=4)	*The HPLC-DAD method described involves extraction of mushroom material with methanol prior to analysis; therefore, it does not allow direct or remote, non-invasive analysis. Off-line chromatographic analysis is chosen because samples are processed externally before insertion into the HPLC system, reflecting typical practice in mycological and toxicological quantification.*	semi-automatic (W=4)	*Instrumental analysis with LC-MS/MS is automated, but sample prep (weighing, extraction) is manual.*	off-line (W=3)	*The GC-FID instrument is lab-based; samples are extracted and brought to the instrument—not measured in-line or on-line at source.*
			none or miniaturized (W=4)	*Sample prep involves sonication (miniaturized extraction) but manual weighing and filtration.*		
Claude	external sample pre- and treatment and batch analysis (reduced number of steps) (W=2)	*The method requires extraction with methanol (4 cycles), filtration, rotary evaporation, redissolution, ultrasound treatment, and syringe filtration before HPLC analysis. While the number of steps is moderate (not excessive), it's not a direct analysis method and requires sample preparation.*	semi-automatic (W=3)	*The method uses automated Desaga Applicator for sample application and TLC scanner with proQuant software for quantitative evaluation. However, extraction (Soxhlet), sample preparation, plate development, and manual handling of plates require significant operator intervention. The densitometric scanning is automated, but overall workflow requires substantial manual operation.*	off-line (W=2)	*The GC-FID analysis is performed off-line. Samples must be collected, prepared in the laboratory (dissolved, diluted, internal standard added), and then analyzed. There is no in-line or at-line measurement capability. This requires transportation and storage of samples before analysis.*
			not miniaturized (W=3)	*Sample preparation involves traditional Soxhlet extraction (5 hours with 50 mL methanol for 5 g sample), concentration steps, and volumetric flask dilutions (25 mL). These are conventional-scale*		

				operations. While the final spotting volume is small (microliters), the upstream preparation is not miniaturized. Modern alternatives like microextraction techniques (SPME, LPME) are not employed.		
Gemini	external sample pre- and treatment and batch analysis (large number of steps) (W=1)	*The procedure is not direct. It requires numerous sample preparation steps before analysis, including methanol extraction, ultrasound assistance, multiple filtration stages, and drying with a rotary evaporator, all performed off-line in a laboratory setting.*	semi-automatic (W=4)	*The procedure is a mix of manual and automated steps. The lengthy sample extraction is manual. Sample application to the HPTLC plate is done with a Desaga Applicator, which is a semi-automated device, and the final densitometric scan is automated and controlled by software.*	off-line (W=2)	*The analysis is performed on a laboratory instrument (Agilent 6890 Network GC System) that is physically separate from the sample's origin. The samples are prepared and then transported to the instrument for analysis, which is characteristic of an off-line process. In this context of quality control, the accuracy of an off-line lab method is prioritized over the speed of an in-situ measurement.*
			not miniaturized (W=4)	*The sample preparation is not miniaturized. It uses a substantial amount of solid sample (5 g) and solvent (50 mL) in conventional laboratory glassware.*		
